# Highly Dispersed NiO Nanoparticles Decorating graphene Nanosheets for Non-enzymatic Glucose Sensor and Biofuel Cell

**DOI:** 10.1038/srep36454

**Published:** 2016-11-02

**Authors:** Guisheng Zeng, Weiping Li, Suqin Ci, Jingchun Jia, Zhenhai Wen

**Affiliations:** 1 Key Laboratory of Jiangxi Province for Persistent Pollutants Control and Resources Recycle, Nanchang Hangkong University, Nanchang 330063, P. R. China; 2 Key Laboratory of Design and Assembly of Functional Nanostructures, Fujian Institute of Research on the Structure of Matter, Chinese Academy of Sciences, Fuzhou, Fujian 350002, P. R. China; 3 Fujian Provincial Key Laboratory of Nanomaterials, Fujian Institute of Research on the Structure of Matter, Chinese Academy of Sciences, Fuzhou, Fujian 350002, P. R. China

## Abstract

Nickel oxide-decorated graphene nanosheet (NiO/GNS), as a novel non-enzymatic electrocatalyst for glucose oxidation reaction (GOR), was synthesized through a facile hydrothermal route followed by the heat treatment. The successful synthesis of NiO/GNS was characterized by a series of techniques including XRD, BET, SEM and TEM. Significantly, the NiO/GNS catalyst show excellent catalytic activity toward GOR, and was employed to develop a sensitive non-enzymatic glucose sensor. The developed glucose sensor could response to glucose in a wide range from 5 μM–4.2 mM with a low detection limit (LOD) of 5.0 μM (*S*/*N* = 3). Importantly, compared with bare NiO, the catalytic activity of NiO/GNS was much higher. The reason might be that the 2D structure of graphene could prevent the aggregation of NiO and facilitate the electron transfer at electrode interface. Moreover, the outstanding catalytic activity of NiO/GNS was further demonstrated by applying it to construct a biofuel cell using glucose as fuel, which exhibited high stability and current density.

Since Clark and Lyons reported the first biosensor based on enzyme electrode in 1962, tremendous effort has been directed toward research in developing high-performance glucose enzyme biosensors due to their potential application in various fields, such as medical diagnosis, diabetes management, bioprocess monitoring, beverage industry, and environmental monitoring^1^. The traditional enzyme-based glucose biosensor are typically immobilized the glucose oxidase on various substrates including silica^2^, TiO_2_ nanotube arrays^3^, carbon nanotubes (CNTs)^4^, Au and ZnO etc.^5^,^6^,^7^,^8^. Although enzyme based glucose sensors have advantage of high sensitivity and selectivity, the activity and stability of enzyme are highly dependent on environmental conditions, such as pH, temperature and other factors^9^. Therefore, it is highly desirable to explore non-enzymatic electrocatalyst with high activity and excellent stability for catalyzing glucose oxidation.

Recently, a variety of noble metal based materials, such as Pt-CNT^10^, Pd-SWCNT^11^, MWCNT-RuO_2_^12^, Au-Pt alloy^13^, Pt-Ir alloy^14^ etc., have been investigated as electrocatalysts of GOR for developing biosensor or biofuel cell device. However, these electrocatalytic materials are noble and rare metal with expensive price; moreover, they are vulnerable to the chemisorbed intermediates and adsorbed chloride ion^15^. In recent years, the transitional metal (e.g. Fe, Co and Ni) compounds have received intensive research interests as cost effective elecctrocatalysts for GOR^16^,^17^,^18^,^19^,^20^,^21^,^22^,^23^. Among them, Ni-based material is one of the most competitive candidates because of their low toxicity, low price, good stability, and high catalytic activity toward GOR. Unfortunately, the poor electrical conductivity of Ni-based materials increases both the sheet resistance and the charge transfer resistance of the electrode, which in work reported to date have led to a poor catalytic activity or sensing performance^24^. Therefore, carbon nanomaterials, such as CNT^19^, activated carbon and graphene^25^, are usually designed to be the supports of these poor conductive material to enhance their conductivity and improve the effective contact area. For instance, graphene-cobalt oxide^26^ and nickel oxide on CVD-grown graphene^27^ have been used for non-enzymatic glucose sensor. Due to extraordinary electrical and physicochemical properties, graphene has become one of the most competitive additives employed to advance the functionality of Co, Ni-based compound. Besides, the introduction of graphene also bring lots of active sites that tightly attaching metal oxide nanoparticles, which potentially provide additional advantage to prevent the agglomeration of active material during their catalytic process and thus are more stable and practical upon application.

In this paper, we reported the fabrication of graphene nanosheets supported highly dispersed NiO nanoparticles (NiO/GNS) via a simple and convenient hydrothermal method. Electrochemical tests demonstrated that the NiO/GNS nanostructures exhibit excellent catalytic activity and high selectivity toward GOR, directing us to develop a high-performance non-enzyme glucose biosensor. In addition, to our best knowledge, this is the first demonstration of a low cost and enzymeless glucose biofuel cell device with the NiO/graphene as the electrode material.

## Experimental

### Chemicals and reagents

Graphene oxide hydrosol (Chinese Academy of Sciences, Shanxi Institute of Coal Chemistry); All other reagents, purchased from Xilong Chemical and Hefei Bomei Biological Technology, were of analytical grade and used as received without further purification. All solutions were freshly prepared with Milli-Q deionized (DI) water.

### Material synthesis

Typically, the NiO/GNS materials were prepared as follows: 0.1 g sodium dodecyl sulfate (SDS) was dissolved in 30 mL of graphene oxide (GO) aqueous solution (5.0 mg mL^−1^) at room temperature. Then, 5.0 mmol (1.19 g) nickel chloride hexahydrate (NiCl_2_•6H_2_O) were added into the solution with vigorous magnetic stirring until they are completely dissolved. Afterwards, 30 mL of ethanol and 5.0 mmol (0.3 g) of urea were added into the solution with vigorous magnetic stirring. After stirring for 10 minutes, the mixed solution were transferred to a 100 mL Teflon-lined stainless steel autoclave and heated at 160 °C for 10 h. After cooling to room temperature, the products were filtered and washed with distilled water and absolute alcohol for three times, respectively. The dried black powder was annealed at 500 °C for 5 h in Ar flow, with a heating rate of 5 °C min^−1^. The product was named nickel oxide/graphene nanosheets (NiO/GNS). For comparison, the bare NiO samples were prepared in accordance with the above procedure without GO solution.

### Material characterization

Powder X-ray diffraction (XRD) was conducted on a D8 ADVANCE (Bruker) powder diffractometer. The structure and morphology of the samples were characterized using a scanning electron microscope (Nova Nano SEM450, FEI) and a transmission electron microscopy (TEM, JEM-2010). Specific surface area, pore volume and pore size distributions were tested at 77.3 K through Brunauer-Emmett-Teller (BET, Quantachrome Nova) nitrogen adsorption-desorption.

### Electrode preparation and electrochemical measurement

To prepared the working electrode, 5 mg electrode materials (NiO/GNS or NiO) and 0.05 mL Nafion solution (DuPont) were added into 0.45 mL water, then sonicated for 30 min and formed a homogeneous suspension. The electrode was prepared by dripped 6.0 μL of the suspension onto glassy carbon electrode (GCE, diameter 3 mm, polished with 0.05-micron alumima paste); the modified electrode after drying was then used as the working electrode. Cyclic voltammetry and current-time curve data were recorded by an electrochemical workstation (CHI 660D), using a Pt wire as the counter electrode and Ag/AgCl (saturated KCl) as the reference electrode. Before the measurement, the 0.1 M NaOH aqueous solution was purged with Ar for ~15 mins to remove the dissolved oxygen. For fabricating glucose fuel cell, a slurry was prepared by mixing the NiO/GNS sample, acetylene black, and polyvinylidene fluoride (PVDF) at a weight ratio of 80:15:5, using N-methyl-2-pyrrolidone (NMP) as a solvent; the mixture was then uniformly coated on the carbon cloth (1.5 cm × 2 cm) with a mass loading of about 5.0 mg cm^−2^. The cathode electrode was prepared by the same method using the commercial Pt/C (20 wt%) instead of NiO/GNS. A batch-type microbial fuel cell (MFC) with two chambers was constructed by connecting two glass bottles (25 mL, Phychemi, Beijing) with an anion-exchange membrane (AEM, Membrane International Inc., Ringwood, NJ, USA) as a separator. The cell voltage was recorded by a digital multimeter (Keithley Instruments, Inc.).

## Results and Discussion

### Characterization

**Figure 1:**
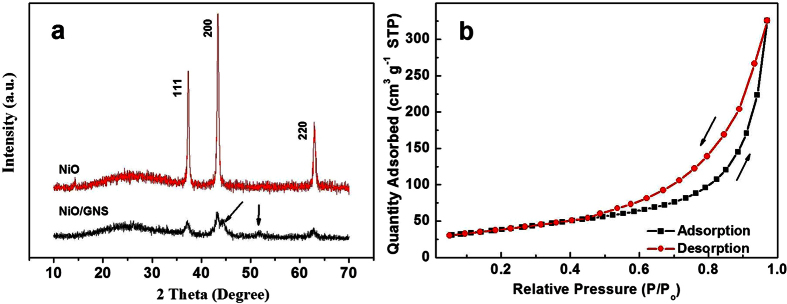
(**a**) XRD patterns of NiO and NiO/GNS. (**b**) The BET image of NiO/GNS.

The X-ray diffraction analysis was performed to characterize the samples on a small panel. Figure 1a shows the typical powder XRD pattern of the bare NiO and the NiO/GNS nanoparticles. Both samples exhibits three prominent peaks at the 2 Theta value of 37.3°, 43.4° and 62.9°, which can be well indexed as (111), (200) and (220) crystal planes of the NiO phase (JCPDS NO. 01-078-0429), respectively. The results showed that Ni(OH)_2_ has been completely converted to a pure phase NiO after annealing at 500 °C under Ar atmosphere. Addition of graphene made the diffraction peaks weaker and broader, suggesting the smaller crystallite sizes of NiO nanoparticles in NiO/GNS as compared with the bare NiO. The two new weak peaks at 44.9° and 51.8° observed over the NiO/GNS that are assigned to face-centered-cubic (fcc) Ni since the graphene nanosheets can probably induced partly reduction of the NiO nanoparticles anchored on the graphene nanosheets^28^. Figure 1b shows the nitrogen adsorption-desorption isotherm for NiO/GNS, which exhibited the type IV isotherms with a distinct hysteresis loop at a relative pressure P/P_0_ ranging from 0.5 to 1.0. According to the IUPAC nomenclature, this curve is a typical difference between the adsorption and desorption of mesoporous material. The shape of the hysteresis loop is associated with silt-like pores formed by the aggregations of NiO nanoparticles, suggesting the sample is composed of sheet-like graphene decorated with NiO^29^. The as-prepared NiO/GNS samples possess a BET surface area of 135.8 m^2^/g, a pore volume of ~0.504 cm^3^/g, and an average pore size of ~14.8 nm.

**Figure 2:**
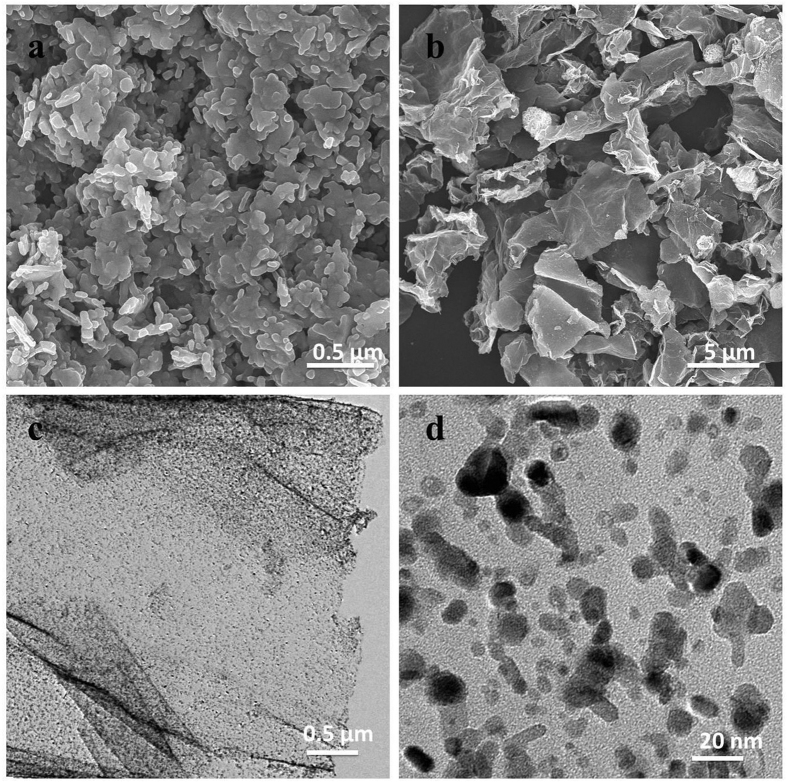
(**a**,**b**) Typical FESEM images of NiO and NiO/GNS. (**c**,**d**) TEM images of NiO/GNS with different magnification.

The morphology and structure of the NiO and NiO/GNS samples were examined by SEM and TEM. Figure 2a is the SEM image of the bare NiO sample, indicating the NiO nanoparticles had a nanorod morphology with a diameter of around 60 nm and a length ranging of 200–400 nm; they are agglomerated together forming a block product. Figure 2b displays the typical SEM image of NiO/GNS, revealing the composites have a nanosheets morphology and crumpled structure on graphene surface, probably arising from the hydrothermal treatment. The NiO nanoparticles (white dots) are uniformly dispersed on graphene surface, demonstrating that graphene plays a key role in producing smaller NiO nanoparticles and preventing the agglomeration of NiO nanoparticles. For TEM images of the NiO/GNS (Fig. 2c), one can observe a large amount of nanoparticles uniformly anchored on the graphene surface. In addition, Fig. 2d shows the high resolution TEM (HRTEM) image, demonstrating that the NiO has a nanoparticle size ranging from 15 nm to 30 nm and strongly anchored on the surface of graphene nanosheets.

### Electrocatalysis of glucose oxidation

**Figure 3:**
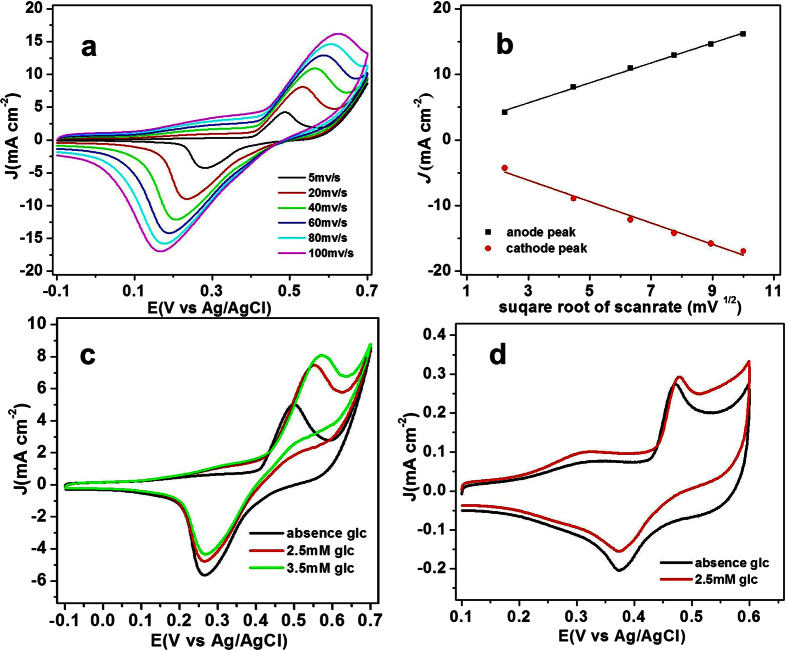
(**a**) CVs of the NiO/GNS in 0.1 M NaOH at different scan rates (5–100 mV s^−1^). (**b**) Relationship between J and v^1/2^ for CVs of the NiO/GNS in 0.1 M NaOH. (**c**) CVs of the NiO/GNS in 0.1 M NaOH in the absence and presence of glucose at a scan rate 10 mVs^−1^. (**d**) CVs of the NiO in 0.1 M NaOH in the absence of glucose and the presence of glucose at a scan rate of 10 mVs^−1^.

The electrochemical properties of NiO/GNS were firstly investigated using CV technique in Ar-saturated 0.1 M NaOH solution at various scan rate (5~100 mV s^−1^) as shown in Fig. 3a. A couple of redox peaks for the NiO/GNS are observed at the potential from −0.1 to 0.7 V for NiO/GNS electrode, which could be attributed to the redox reaction between NiO and NiOOH, respectively^24^. In addition, the anodic peak shows a slight positive shift accompanying with negatively moving of the cathodic peak with the increase of scan rate, which is consistent with peak potentials shift toward the formal potential as period is increased for the quasi-reversible mechanism, suggesting a quasi-reversible electron transfer reaction for the above electrochemical reaction^30^. Additionally, Fig. 3b shows the relationship between the peak current density and the square root of the scan rate, the peak current densities for both the oxidation and reduction is proportional to the square root of the scan rate, suggesting that the electrochemical reaction occurring on the surface of the NiO/GNS is a diffusion-controlled process^31^.

The electrocatalytic activity of NiO/GNS toward GOR was studied in 0.1 M NaOH solution with the presence of a certain amount of glucose. Figure 3c presents the CVs in the absence and the presence of 2.5 mM and 3.5 mM glucose in 0.1 M NaOH at the NiO/GNS electrode with a scan rate of 10 mV s^−1^. As expected, the anodic peak current increased with addition of glucose, and the peak intensity enhanced with the increase of glucose concentration. The addition of glucose causes remarkable increase in anodic peak current, while the cathodic peak current slightly decreased. And the glucose is electrocatalyzed into glucolactone at the NiO/GNS electrode by the NiO/NiOOH redox couple according to the following reactions^32^:


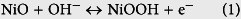



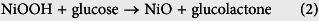


The remarkable increase of the anodic peak current demonstrates the NiO/GNS/GCE has strongly electrochemical response upon glucose oxidation. As described in the equation (1) and (2), the catalytic process is accompanied with the redox reaction between divalent Ni and trivalent Ni. Therefore, it is reasonable that the reduction current decrease slightly with the addition glucose due to the consumption of the trivalent Ni by glucose oxidation. Figure 3d shows the CV responses in the absence of glucose and the presence of 2.5 mM glucose. According to the experimental results, the bare NiO exhibits a much smaller current response to the same amount of glucose as compared with the NiO/GNS electrode, demonstrating that the NiO/GNS significantly improved the catalytic activity on glucose oxidation than that of bare NiO due to improved conductivity (Fig. S1).

**Figure 4:**
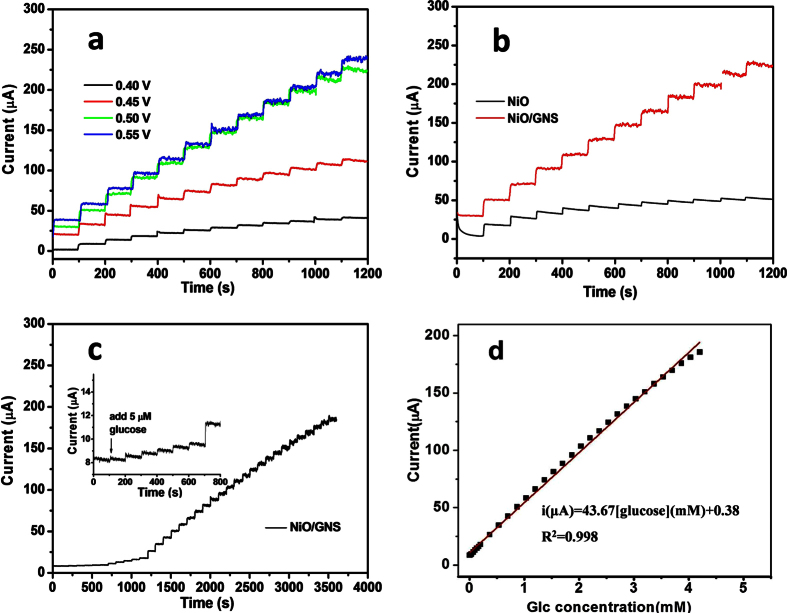
(**a**) Amperometric response of the NiO/GNS with successive addition of 0.5 mM glucose into 0.1 M NaOH solution at different potentials. (**b**) Amperometric response of the NiO and the NiO/GNS with the successive addition of 0.5 mM glucose at 0.50 V. (**c**) Amperometric response of the NiO/GNS to successive addition of glucose at 0.5 V; inset: amperometric response to 5.0 μM glucose. (**d**) The corresponding calibration curve at the NiO/GNS eletrode.

Since the applied potential is one of the key factors in affecting the electrochemical signal response and realizing selective determination of glucose, it is thus of great importance to conduct comparison experiment to optimize the working potential of glucose electrochemical sensor. Figure 4a exhibits the amperometric response of the NiO/GNS electrode to successive additions of 0.5 mM glucose at different applied potentials. The steady-state current response increased drastically when the applied potential was tuned from 0.40 V to 0.55 V upon addition of glucose. However, when the potential rose to 0.55 V, the amperometric response did not increase significantly. Given that the lower potential tend to result in a smaller background current or noise, while a higher potential may induce interrupt signal from a lot of interference species, 0.50 V was selected as the optimum working potential for the amperometric measurement in the later studies. It should be noted that the bare NiO only shows a slight current response with addition of glucose, much poorer than that of the NiO/GNS (Fig. 4b), which is basically consistent with the behavior observed in the CV results (Fig. 3d).

The sensitivity of the NiO/GNS electrode toward glucose was evaluated by amperometric measurements at a constant potential of 0.50 V. Figure 4c shows the typical amperometric response of the NiO/GNS electrode to the successive step-wise addition of glucose into the stirring electrolyte (0.1 M NaOH) with a time interval of around 100 s. When the concentration of glucose raise to 5.0 μM, the NiO/GNS electrode shows an observable response and achieve the maximum steady-state current within 5s (the inset Figure in Fig. 4c, partially enlarged figure), implying the catalytic reaction of glucose oxidation is promptly activated on the surface of the NiO/GNS electrode. Figure 4d presents the calibration plot of glucose concentration and the corresponding current density, demonstrating the NiO/GNS electrode shows a good linear relationship over a wide concentration range of 5.0 μM–4.2 mM glucose with a slope of 46.67 μA mM^−1^ and a correlation coefficient of 0.998. The sensitivity of the NiO/GNS sensor is calculated to be 666.71 μA mM^−1^ cm^−2^ by dividing the slope of the linear regression equation by the electrode surface area, this value stands on the medium level relating to the previously reported graphene or the NiO-based glucose biosensors^24^,^31^,^33^,^34^,^35^. Additionally, based on a signal-to-noise ratio of 3 (S/N), a lower detection limit of 5.0 μM can be obtained.

### Interference study and long-term test

**Figure 5:**
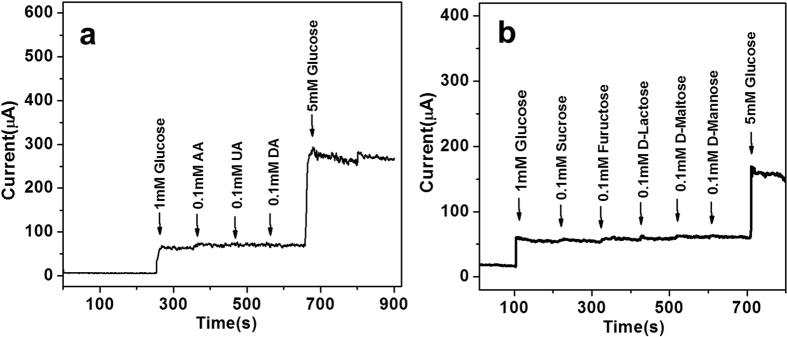
(**a**) Amperometric response of the NiO/GNS to successive addition of 0.1 mM AA, 0.1 mM UA, 0.1 mM DA to glucose at an applied potential of 0.50 V. (**b**) Amperometric response of the NiO/GNS to successive addition of 0.1 mM Sucrose, Fructose, D-Lactose, D-maltose, D-Mannose to glucose aqueous solution.

The interference in nonenzymatic glucose sensor is a big challenge because a few of endogenous interfering species, such as ascorbic acid (AA), dopamine (DA), and uric acid (UA), usually co-exist with glucose in human blood^36^. And the level of glucose (3–8 mM) is much higher than these species (<0.5 mM)^37^. Therefore, we carried out interference study by adding 1.0 mM glucose in electrolyte during amperometric measurement at a constant potential of 0.50 V, followed by successively adding 0.1 mM AA, 0.1 mM UA, and 0.1 mM DA to mimic the interference. As shown in Fig. 5a, the NiO/GNS exhibits excellent selectivity for GOR, the corresponding oxidation current change is 56.2 ± 0.3 μA upon adding 1 mM glucose, which greatly exceeds those recorded for the interfering species, i.e. 6.6 ± 0.2 μA for DA, and almost no current response upon adding UA and AA. In addition, the selectivity of the NiO/GNS sensor towards GOR was also tested in the presence of a set of sugars. Figure 5b shows that the corresponding oxidation current response for these sugars is relatively small in comparison with the 5 mM glucose, indicating the NiO/GNS electrode has high selectivity for glucose. These results demonstrate that the NiO/GNS modified electrode has a good selectivity for GOR and is a potential material of nonenzymatic glucose sensor.

The long-term stability of the NiO/GNS sensor was evaluated through the amperometric response of 0.2 mM glucose recorded at intervals over 18 days, and the NiO/GNS electrode was stored in refrigerator when not in use. The results indicate that the sensor retains more than 88% of the initial sensitivity in the long-term tests (Figure S2), suggesting that the non-enzymatic glucose sensor has favorable stability. This excellent durability mainly results from the robust mechanical stability of 2D graphene.

### Glucose fuel cell

**Figure 6:**
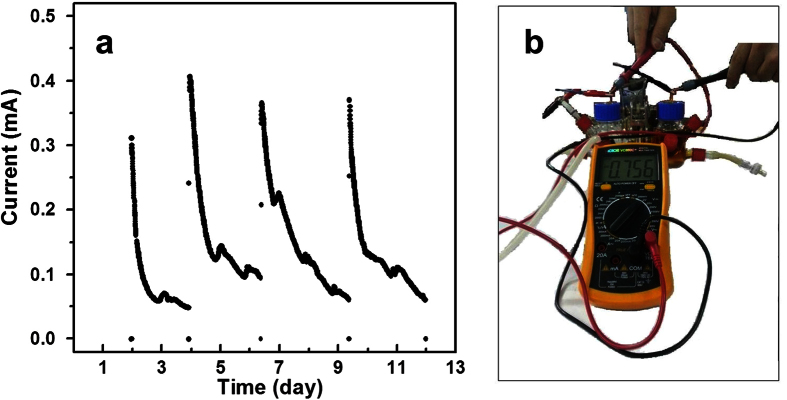
(**a**) The electrochemical oxidation of NiO/GNS||Pt/C fuel cell, using 0.1 M KOH solution and 0.1 M KOH solution containing 0.1 M glucose as catholyte and anolyte. (**b**) the schematic diagram of fuel cell test for open circuit voltage.

Since the NiO/GNS shows excellent catalytic activity towards GOR, a non-enzyme glucose fuel cell was fabricated by using the NiO/GNS as anode and the commercial Pt/C as cathode. The cell tests were carried out in a batch-type MFC with two chambers separated by an anion-exchange membrane. The anolyte is 0.1 M KOH aqueous solution containing of 0.1 M glucose, and the catholyte is 0.1 M KOH aqueous contained dissolved oxygen using a small air pump (shown in Fig. 6b). The open-circuit voltage of the fuel cell reached a relatively considerable voltage up to 0.756 V^38^. As is shown in Fig. 6a, during every running process the current decreased accordingly with the consumption of glucose with loading of a 100 ohm resistor. However, the current recovered instantly when the electrolyte was refreshed. After four cycles the initial currents of cell still maintained at around 0.3 mA (corresponding current density: 0.66 A m^−2^), also indicating the high stability of the electrode in catalytic reaction.

## Conclusions

In this paper, we developed a reliable strategy to fabricate a composites of highly dispersed nickel oxide nanoparticles decorated graphene nanosheets (NiO/GNS), as an excellent electrocatalysis toward glucose oxidation reaction (GOR). The synthesized NiO/GNS shows fascinating physical and chemical features with mesoporous structure, high surface area, high conductivity, and robust cycle stability. Compared with the bulk NiO, the NiO/graphene shows significantly improved (two times higher) activity toward catalyzing glucose oxidation reaction. The high catalytic activity of NiO/GNS can be attributed to highly dispersed NiO nanoparticles and enhanced electron transfer. Inspiringly, the NiO/GNS electrochemical glucose sensor could detect glucose with high sensitivity, possibly providing a low-cost, stable and non-enzymatic point-of-care diagnostics tool for glucose monitoring. Furthermore, the NiO/graphene was applied as the electrode material of an enzymeless glucose biofuel cell for the first time. The enhanced performance of the NiO/GNS biofuel cell can potentially pave the way of powerful catalyst for energy conversion.

## Additional Information

**How to cite this article**: Zeng, G. *et al*. Highly Dispersed NiO Nanoparticles Decorating graphene Nanosheets for Non-enzymatic Glucose Sensor and Biofuel Cell. *Sci. Rep.* **6**, 36454; doi: 10.1038/srep36454 (2016).

**Publisher’s note**: Springer Nature remains neutral with regard to jurisdictional claims in published maps and institutional affiliations.

## Supplementary Material

Supplementary Information
