# Supplementary material for: Highly Dispersed NiO Nanoparticles Decorating graphene Nanosheets for Non-enzymatic Glucose Sensor and Biofuel Cell
Source: Sci Rep. 2016 Nov 2;6:36454. doi: 10.1038/srep36454 (PMC5090366; doi:10.1038/srep36454)
Supplement: Supplementary Information [file srep36454-s1.pdf]

Supporting Information:

Highly Dispersed NiO Nanoparticles Decorating graphene Nanosheets for Non-enzymatic Glucose Sensor and Biofuel Cell

Guisheng Zeng,[1] Weiping Li,[1,2] Suqin Ci,[1] Jingchun Jia,[2,3] Zhenhai Wen,[1,2,3*]

1 Key Laboratory of Jiangxi Province for Persistent Pollutants Control and Resources Recycle, Nanchang Hangkong University, Nanchang 330063, PR China

2 Key Laboratory of Design and Assembly of Functional Nanostructures, Fujian Institute of Research on the Structure of Matter, Chinese Academy of Sciences, Fuzhou, Fujian 350002, P. R. China

3 Fujian Provincial Key Laboratory of Nanomaterials, Fujian Institute of Research on the Structure of Matter, Chinese Academy of Sciences, Fuzhou, Fujian 350002, P. R. China

E-mail: wenzhenhai@yahoo.com or wen@fjirsm.ac.cn

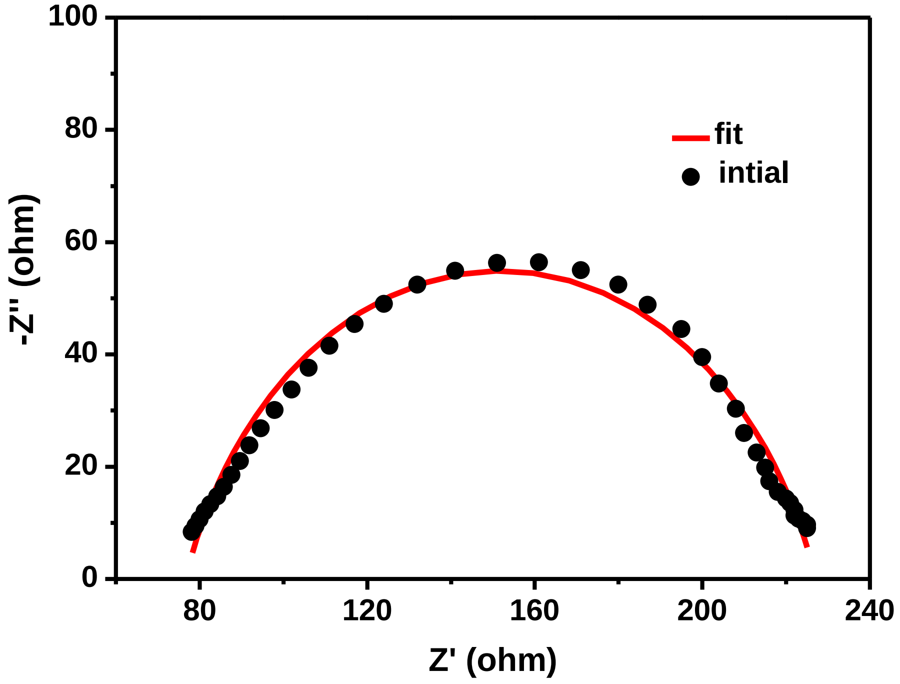

Figure S1. (a) Electrochemical impedance spectra of NiO/GNS electrode in 0.1 M KOH.

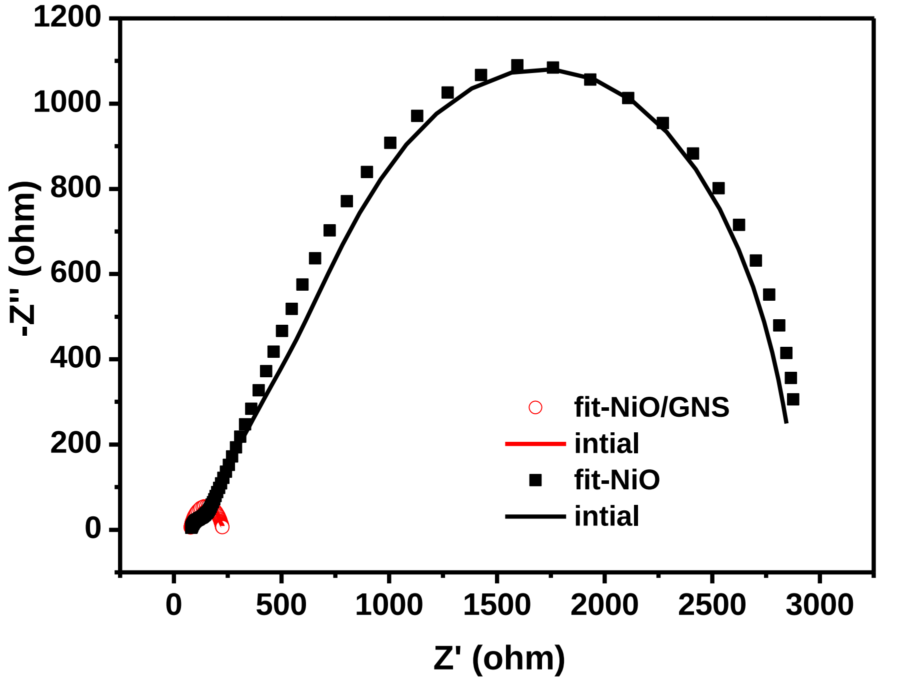

Figure S1. (b)Electrochemical impedance spectra of NiO/GNS and NiO electrodes in 0.1 M KOH.

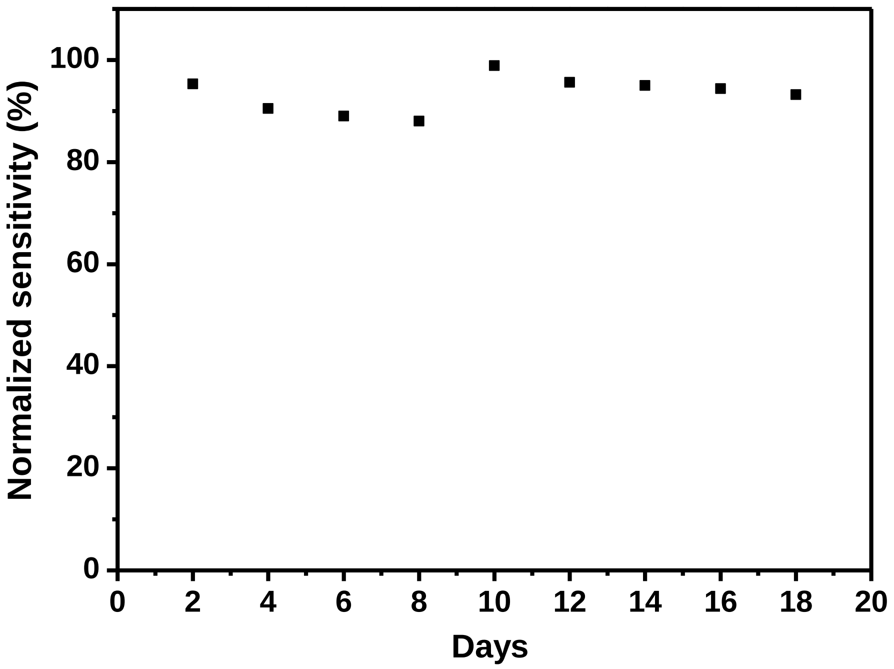

Fig. S2. Normalized sensitivity of the NiO/GNS sensor toward glucose analysis by amperometric measurements for 18 days.

The long-term stability of the NiO/GNS sensor was evaluated through the amperometric response of 0.2 mM glucose recorded at intervals over 18 days, and the NiO/GNS electrode was stored in refrigerator when not in use. The results indicate that the sensor retains more than 88% of the initial sensitivity in the long-term tests (Figure S2), suggesting that the non-enzymatic glucose sensor has favorable stability. This excellent durability mainly results from the robust mechanical stability of 2D graphene.
